# Delineating the nexus between gut-intratumoral microbiome and osteo-immune system in bone metastases

**DOI:** 10.1016/j.bonr.2024.101809

**Published:** 2024-10-10

**Authors:** Shreya Kapoor, Muskan Gupta, Leena Sapra, Taranjeet Kaur, Rupesh K. Srivastava

**Affiliations:** Department of Biotechnology, All India Institute of Medical Sciences (AIIMS), New Delhi 110029, India

**Keywords:** Gut microbiota, Bone metastases, Bone cancer, Immune system, Osteo-immuno-oncology, Biotics, Intratumoral microbiota

## Abstract

Emerging insights in osteoimmunology have enabled researchers to explore in depth the role of immune modulation in regulating bone health. Bone is one of the common sites of metastasis notably in case of breast cancer, prostate cancer and several other cancer types. High calcium ion concentration and presence of several factors within the mineralized bone matrix including TGF-β, BMP etc., aid in tumor growth and proliferation. Accumulating evidence has substantiated the role of the gut-microbiota (GM) in tumorigenesis, further providing a strong impetus for the growing “immune-cancer-gut microbiota” relationship. Recent advancements in research further highlight the importance of the intra-tumor microbiota in conjunction with GM in cancer metastasis. Intratumoral microbiota owing to their ability to cause genetic instability, mutations, and epigenetic modifications within the tumor microenvironment, has been recognized to affect cancer cell physiology. The host microbiota and immune system crosstalk shapes the innate and adaptive arms of the immune system, which is the key player in cancer progression. In this review, we aim to decipher the role of microorganisms mediating bone metastasis by shedding light on the immuno-onco-microbiome (IOM) axis. We discussed the feasible cancer therapeutic interventions based on the modulation of the microbiome-immune cell axis which includes prebiotics, probiotics, and postbiotics. Here, we leverage the conceptual framework based on the published articles on microbiota-based therapies to target bone metastases. Understanding this complicated nexus will provide insights into fundamental factors governing bone metastases which will subsequently help in managing this malignancy with better efficacy.

## 1 Introduction

The majority of deaths due to cancer are a result of incurable metastases, which occur when genetically unstable cancer cells disseminate to a site distant from the primary tumor (Fares et al., 2020; Mani et al., 2024). The roaming tumor cells interact with their receptive target tissues, but colonization occurs only at particular organ sites, which provides a microenvironment that is conducive to the growth and proliferation of tumor cells (Valastyan and Weinberg, 2011). Disseminated tumor cells (DTCs) are believed to remain dormant even after migrating to distant organs until an unknown trigger drives them to interact favorably with the new environment to obtain an advantage for growth and survival (Fares et al., 2020).

Bone is one of the common sites for the metastasis of solid tumors (Coleman et al., 2020). Estimates from the available literature suggest that the highest incidence of metastatic bone disease is observed in cancers arising from the breast (75 %) (Zhong et al., 2023) and prostate (70 %) (Zhou et al., 2024), and non-small-cell lung cancer (20–50 %) (Gong et al., 2024), kidney (40 %) (Coleman et al., 2020) and thyroid (∼40 %) (Yao et al., 2024). The bone's unique microenvironment characterized by high abundance of calcium ions and the chemo-attractiveness fueled by the release of growth factors from the bone matrix and prostaglandins or other factors from different bone cells, including stromal cells, osteoblasts, osteocytes, and osteoclasts, make it an excellent place for tumor cells to colonize (Coleman et al., 2010, Coleman et al., 2020; Elaasser et al., 2024). Intriguingly, bone is a unique environment that contains a wide range of immune cells and bone cells. These cells are known for their reciprocal relationships with the tumor and bone-resident cells, which facilitate the effective colonization of DTCs in the bone (Coleman et al., 2010; Ryan et al., 2022). Hence, this crosstalk between the bone and immune system is well established which further discerns the interaction of immune cells and cancer cells during bone metastases.

The role of gut microbiota (GM) in shaping both the adaptive and innate arms of the immune system has been mapped which further sheds light on the immuno-onco-microbiome (IOM) axis to derive insights into the microbial population modulating tumorigenesis (Sepich-Poore et al., 2021). However, it is unclear whether the bacteria inhabit a hospitable microenvironment within the tumor to multiply, or whether they are the main agents of carcinogenesis and are thus responsible for the persistence of the tumor and its resistance to anticancer therapies. For identifying host-centric tumor characteristics, it is essential to dissect the complex interplay between the GM, anti-tumor activity, and immunosurveillance. Recent research has witnessed a surge in investigations understanding IOM axis (Li et al., 2024b; Sepich-Poore et al., 2021), particularly in malignancies such as colon cancer (Jia et al., 2024a; Zhang et al., 2023a) melanoma (Bender et al., 2023a; Björk et al., 2024; Lee et al., 2023) and breast cancer (Jia et al., 2024a; Lee et al., 2023; Viswanathan et al., 2023). It can thus be hypothesized that the gut may also have direct or indirect effects on secondary cancers, such as secondary bone metastasis. This can thus provide more insights about the relationship between GM and the initiation, progression, prognosis, and treatment of bone cancer. In this review, we comprehensively discuss the likely mechanisms through which GM can regulate the onset and progression of bone cancer and how intra-tumor microbiota can affect the host's response to the therapies. Furthermore, we examine the underlying strengths of modulating the microbial profiles and immune-oncosystem through microbiological interventions to serve as a feasible therapeutic intervention in cancers.

## 2 Bone cancer and metastases

Bone is a rigid yet extremely dynamic, metabolically active organ, that serves a structural purpose by supporting vital internal organs and bone marrow simultaneously providing the site for muscle attachment for movement. Also, bone is a reservoir of minerals and energy that contributes to the maintenance of serum homeostasis via buffering action. To sustain skeletal strength and integrity, bone undergoes a continuous remodeling process within the basic multicellular unit. Resorption, reversal, and formation are the three consecutive steps that constitute the bone remodeling process as reviewed in detail in other reviews (Bolamperti et al., 2022; Han et al., 2018; Wang et al., 2022b). Depending on the site of origin, bone cancers are classified into primary or secondary.

The primary bone malignancies, such as osteosarcoma, chondrosarcoma, and Ewing sarcoma are rare, however, the risk of developing bone metastases in advanced cancers is quite high. Although the incidence of bone metastases varies among different cancer types, primary tumors originating from the breast and prostate have the highest relative incidence of bone colonization (Ibraheem, 2022; Macedo et al., 2017).

### 2.1 Primary bone cancer

Primary bone tumors are a rare and heterogeneous group of mesenchyme-derived neoplasms that account for only 0.2 % of all human neoplasia (Franchi, 2012; Sung et al., 2021). The latest fifth edition of the World Health Organization (WHO) classifies bone tumors into cartilage tumors, osteogenic tumors, fibrogenic tumors, vascular tumors of bone, osteoclastic giant cell-rich tumors, notochordal tumors, hematopoietic neoplasms of bone, and other mesenchymal tumors of bone (Choi and Ro, 2021). These tumors emerge due to close interactions with the cells of the local bone microenvironment including osteoblasts, osteoclasts, macrophages, and tumor-infiltrating lymphocytes, etc. (Heymann et al., 2019). Three commonly known subtypes of primary bone cancers – osteosarcoma, Ewing sarcoma, and chondrosarcoma – are briefly reviewed below (Brown et al., 2018).

Osteosarcoma (OS) is a highly malignant bone tumor and the third most common cancer in adolescents worldwide, with an OS incidence of 3.4 per million people per year (Mirabello et al., 2009). Due to the sporadic origin of OS, the majority of cases exhibit features of chromosomal abnormality involving genetic alteration or inactivation of germline tumor protein 53 (TP53), retinoblastoma protein (RB1), RecQ like helicase 4 (RECQL4), bloom syndrome gene (BLM) and Werner syndrome ATP-dependent helicase (WRN) (Martin et al., 2012).

Ewing sarcoma (EWS) is the second most common primary bone tumor affecting children and adolescents. Molecular analysis revealed the presence of a characteristic chromosomal translocation and the resulting EWS-FLI1 gene fusion in nearly 85 % of the examined patients (Balamuth and Womer, 2010). EWS-FLI-1 fusions have been reported to form R-loops that block the replication machinery in cells and consequently lead to high levels of DNA damage (Gorthi et al., 2018). Although much is known about the diverse roles of EWS-FLI-1 in epigenetic remodeling, non-coding RNA regulation, oncogene activation, and tumor suppressor repression, the origin cell of EWS is still unknown (Pachva et al., 2021).

Chondrosarcoma (CS) represents a group of tumors that predominate in the cartilage and range from low-grade to high-grade tumors depending upon their metastatic potential. These tumors account for nearly 10–20 % of all primary malignant bone tumors (Thorkildsen et al., 2019). Perturbations in the Hedgehog signaling pathway, tumor suppressor pathways, and PI3K-Akt-mTOR pathway have been reported to contribute to the development of cartilaginous neoplasms (Chow, 2018). Mutations in the exostosin glycosyltransferase (EXT1 or EXT2) and isocitrate dehydrogenase (IDH) 1 and 2 genes are also found in such tumors (Amary et al., 2011).

### 2.2 Secondary bone cancer

Advanced-stage tumor with their origins mapped to the breast, prostate, and lung have a fairly high probability of metastasizing to the bone (Table 1). In body, cancers spread to the bone marrow via the hematogenous or lymphatic system (Coleman, 2001; Coleman et al., 2010).

**Table 1:** Cancers with high potential for bone metastasis and their respective tumor microbiome.

Cancer type	% Bone metastasis	Underlying mechanisms for bone metastases	Tumor microbiome profile	References
Breast cancer	∼75 %	RANKL-dependent and independent osteoclastic differentiation of HSCs	*Pseudomonas*, *Proteus*, *Sphingomonas yanoikuyae*, *Enterococcus*, *Cladosporium*, *Lactobacillus*, *Methylobacterium radiotolerans*, *Streptococcus*	(Fu et al., 2022; Lucas et al., 2018; Ma et al., 2020b; Tzeng et al., 2021)
		Parathyroid hormone-related protein (PTH-rP)		
		connexin-43 gap junctions mediated regulation of intracellular Ca concentrations		
		heterotypic adherent junctions (E-cadherin and osteogenic N-cadherin and OB-cadherin)		
Prostate Cancer	∼70 %	PCa cells secrete OB-stimulating GFs: ET-1, adrenomedullin, FGFs, PDGFs, and BMPs	*Cutibacterium acnes*, *Staphylococcus aureus*, *Listeria Monocytogenes*, *Xanthomonas albilineans*, *Pseudomonas*, *Escherichia*, *Acinetobacter*	(Jiang et al., 2023; Kustrimovic et al., 2023; Ma et al., 2020a)
		loss of tumor-intrinsic type-I IFN driving cancer cell progression		
		Upregulates RANK-L expression in bone stromal cells		
		Increased Activin A expression and thus expression of osteoblastic lesions		
Lung Cancer	∼25–40 %	Increased EGFR expression and osteolytic factors: PTHrP, IL-11	*Modestobacter*, *Propionibacterium*, *Acinetobacter*, *Cyanobacteria*, *Enterobacteriaceae*, *Klebsiella*, *Veillonella*, *Megasphaera*	(Greathouse et al., 2018; Zhou et al., 2017)
		OCs support DTCs, proliferation via IL-19/IL-20RB/STAT3 axis		
		OBs express SDF-1 that increases MMP-9 expression through CXCR4 mediated activation of MAPK/ERK pathway		
Renal cancer	∼20–40 %	RC cells secrete PTHrP, TGF-β, increase RANKL production	*Blautia*, *Streptococcus*, *Ruminococcus*, *Romboutsia*, *Eubacterium*	(Piao et al., 2023; Umer et al., 2018)
		Bone cells secrete PDGF, FGF, IGF therefore promoting RC cells migration		
		increase expression of calcium sensing receptors		

HSCs: Hematopoietic stem cells, PTH-rP: Parathyroid hormone-related protein, Ca: Calcium, PCa: Prostrate cancer, GFs: Growth factors, ET-1: Endothelin-1, FGF: Fibroblast growth factor, PDGF: Platelet-derived growth factor, BMP: Bone morphogenic protein, IFN: Interferon, RANK-L: Receptor activator for nuclear factor kappa-B ligand, EGFR: Epidermal growth factor receptor, IL- Interleukin, OC: Osteoclast, DTC: Disseminated tumor cells, STAT3: Signal transducer and activator of transcription 3, OB: Osteoblast, SDF-1: Stromal cell derived factor-1, MMP: Matrix metalloproteinase 9, CXCR4: C-X-C Chemokine Receptor 4, MAPK/ERK: Mitogen-activated protein kinases/Extracellular signal-regulated kinase, RC: Renal cancer, TGF: Transforming growth factor, IGF: Insulin growth factor.

Tumor cells predominantly interact with bone marrow stromal cells (BMSCs) via CXCL12 that assist in homing of DTCs to the bone (Elaasser et al., 2024). The unique physicochemical properties of bone – hypoxia, intense vascularization, high local calcium concentrations, abundance of immobilized growth factors and acidosis – provide an optimal niche for DTCs to adapt, colonize, and proliferate, as stated by Stephen Paget's “seed and soil” hypothesis (Paget, 1889, Paget, 1989).

The presence of growth factors like IGF, VEGF aid in invasion, growth and survival of metastatic tumor cells while the calcium ions interact with extracellular calcium receptors on the cancer cells and trigger a cascade of downstream signaling events to promote tumor growth and proliferation (Coleman et al., 2020; Elaasser et al., 2024). DTCs from cancers, seed this fertile environment. Furthermore, a recent study suggests that initiation of bone metastasis is coupled with bone remodeling. It has been demonstrated that pathological fractures increase metastatic colonization around the injury. NG2^+^ Bone marrow mesenchymal stem cells are the key players serving dual purposes of bone remodeling and metastasis. These promote metastasis initiation via N-cadherin-mediated cell-cell interaction (Coleman, 2001; Coleman et al., 2020; Elaasser et al., 2024; Zhang et al., 2023b).

Prior to the dissemination of the tumor cells from the primary site, secretion of some systemic factors and micro vesicles (e.g. exosomes) direct the conversion of incipient metastatic sites into compatible ‘pre-metastatic niches’ (Peinado et al., 2017). This prepares the recipient site to undergo extracellular matrix remodeling, increase vascular permeability, and suppress the immune microenvironment. Upon entering the bone microenvironment, DTCs encounter perivascular niche which is a heterogenous network of blood vessels and perivascular cells that decides their ultimate fate. Several factors promote dormancy of DTCs and their retention in perivascular niche while other facilitate their migration to the endosteal niche (Satcher and Zhang, 2022). Vascular E-selectins stimulate mesenchymal to epithelial transition through Wnt signaling and NG2^+^ cells reinforce dormancy via TGF-beta secretion however, endothelial derived E-selectin stimulates the migration of DTCs to endosteal surface which harbours osteoblasts, MSCs and osteoprogenitors that modulate cancer proliferation via several mechanisms involving mTOR signaling, calcium signaling, epigenomic modification etc., subsequently leading to bone metastasis (Satcher and Zhang, 2022).

Bone metastases can manifest either as osteolytic, osteoblastic, or mixed, depending upon the primary mechanism by which tumor cells alter the normal bone remodeling process (Coleman, 2001; Coleman et al., 2020). Osteolytic metastasis is characterized by a lytic and destructive lesion, primarily mediated by osteoclasts, and is not a direct effect of tumor cells on bone. It is predominantly observed in solid tumors of the breast, thyroid, lung, and kidney wherein the cooperation of several genes is required for the formation of aggressive bone lesions (Macedo et al., 2017). Kang et al. reported that elevated expression of pre-metastatic genes - C-X-C chemokine receptor 4 (CXCR4), matrix metalloproteinase (MMP)-1, connective tissue growth factor (CTGF), fibroblast growth factor (FGF5), interleukin (IL)-11, and osteopontin (OPN) – are collectively responsible for the manifestation of osteolytic breast cancer metastasis (Kang et al., 2003).

Osteoblastic metastasis, also known as sclerotic metastasis, is associated with the production of bone-synthesizing factors by tumor cells, which promote the stimulation and proliferation of osteoblasts, resulting in the deposition of new bone (Macedo et al., 2017). Although the underlying mechanisms of osteoblastic bone metastasis are poorly understood, studies have shown indications of tumor osteomimicry and tumor-induced endothelial-to-osteoblast conversion that may provide insights into this domain. Tumor osteomimicry is defined as the ability of cancer cells to express a bone-specific protein profile, including osteocalcin, OPN, and receptor activator of nuclear factor kappa-Β ligand (RANKL), which induce seeding of cancer cells in bone microenvironment (Macedo et al., 2017). Endothelin-1 (ET-1) (Guise et al., 2003), bone morphogenetic proteins (BMPs) (Sun et al., 2020), and growth differentiation factor 15 (GDF15) (Siddiqui et al., 2022) have been reported as signaling osteoblastic factors for bone metastasis.

## 3 Nexus between bone metastases and the immune system: osteo-immuno-oncology

**Fig. 1:**
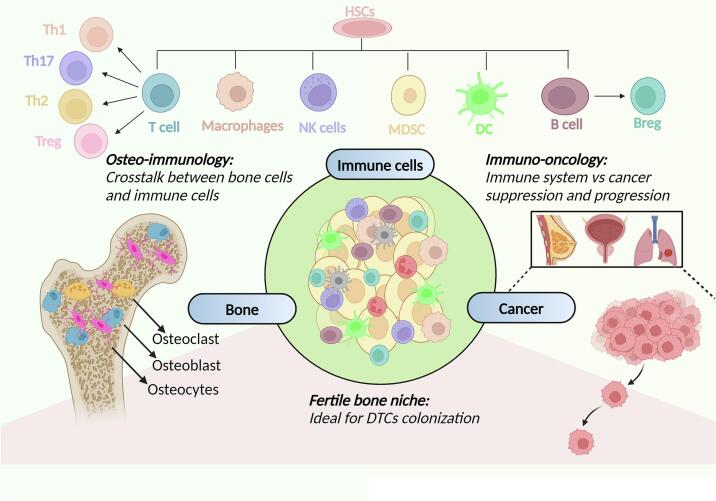
**The osteo-immuno-oncology triad**. Development of immune cells from hematopoietic stem cells (HSCs) in bone marrow indicates that bone and immune system are in constant interaction (Ponzetti and Rucci, 2019). Interaction of bone resident cells (osteoclasts, osteoblasts, osteocytes) with the immune cells is crucial to maintain bone homeostasis. Further, establishment of an immunosuppressive microenvironment and failed anti-tumor immune responses promote bone colonization of tumor cells from advanced cancers of breast, prostate, and lung.

The existence of dynamic cross-communication between the immune system and the skeleton has sparked the emergence of a novel field of immunology known as “Osteoimmunology” (Fig. 1). It specifically deals with the study of the reciprocal regulation of immune cells by the bone cells and their intimate interactions in the bone microenvironment (Guder et al., 2020; Srivastava, 2018). Crosstalk between the skeletal system and immune system is essential for maintaining both osseous homeostasis and immune function. It is well recognized that lymphocytes and immune factors including cytokines, chemokines, and exosomes can promote or antagonize bone remodeling depending upon the external stimuli and the microenvironment as reviewed in detail elsewhere (Guder et al., 2020; Huppert et al., 2022). Now that we can appreciate the mutual dependency of the two systems and the underlying cross-regulation, we are further trying to examine osteoimmune communication in the context of bone metastases.

**Fig. 2:**
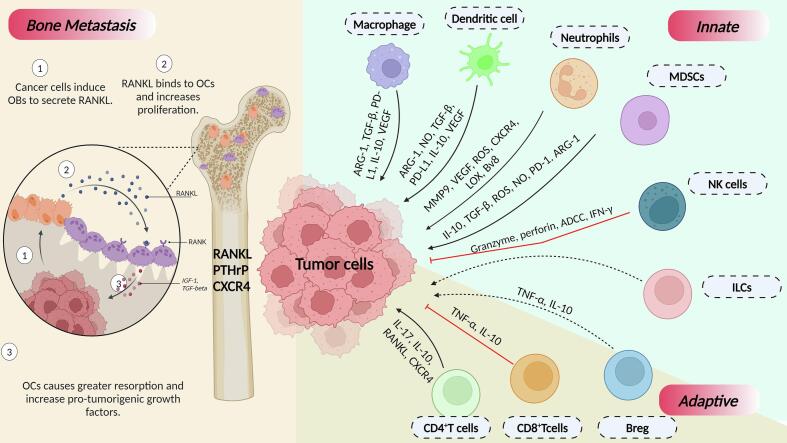
**The interaction between immune cells and tumor cells during bone metastasis**. Under the innate arm of the immune system, macrophages, dendritic cells (DCs), neutrophils, and myeloid-derived suppressor cells (MDSCs) release pro-tumorigenic cytokines that aid in the successful establishment of disseminated tumor cells (DTCs) in bone. Natural killer (NK) cells, however, deploy anti-tumorigenic cytokines that promote tumor regression. On the other hand, CD4 + T cells and Bregs release certain cytokines that stimulate cancer cells to metastasize to bone. Differentiation of CD8 + T cells into cytotoxic T lymphocytes (CTLs) exert anti-tumoral attack by secretion of proinflammatory cytokines- TNF-α and IFN-γ, consequently an anti-metastatic effect. As shown in the figure, the osteolytic tumor cells majorly release pro-osteoclastogenic cytokines such as receptor activator for nuclear factor kappa-B ligand (RANKL), parathyroid hormone-related protein (PTHrP), and C-X-C Chemokine Receptor 4 (CXCR4) which activate osteoclastogenesis and drive formation of osteolytic lesion.

It is an important domain that needs to be addressed particularly because we lack a comprehensive understanding of osteo-immuno-oncology which is essential for developing innovative treatments that specifically target bone metastases. A variety of immune cells including T cells, natural killer cells (Roato and Vitale, 2019), macrophages (Di Mitri et al., 2023) and myeloid-derived suppressor cells (MDSCs) are involved in bone metastases providing an immune-privileged habitat for DTCs to infiltrate and proliferate (Chen et al., 2024) (Fig. 2). Transforming growth factor (TGF)-β-released during osteoclastic bone resorption, promotes tumor growth by interfering with immune defenses because of its inherent immunosuppressive capabilities. In a study by Jiao et al. (2019), TGF-β blockade promotes clonal expansion of CD8^+^ T cells and provide a conducive environment for T cell activation in murine models of bone castrate resistant prostate cancer (Clambey et al., 2012; Jiao et al., 2019).

Treg cells play a crucial role in promoting metastatic tumor growth in the bone microenvironment either by modulating RANK/RANKL axis or by interacting with and suppressing other immune cells including CD8^+^ T cells and helper T cells (Chen et al., 2024). Tregs play a pivotal role in promoting the formation of osteoblastic bone lesions by tilting the balance between osteoclasts and osteoblasts towards osteogenesis by suppressing osteoclast differentiation and proliferation (Huppert et al., 2022). Similar effects have been observed in prostate cancer metastasis to bone wherein increased Treg infiltration often leads to a poor prognosis and increased bone metastasis (Chen et al., 2024). In the immune microenvironment of bone metastasis from prostate cancer, Tregs can translocate to the bone marrow through (CXCR4)/(CXCL12) and can trigger the formation of osteoblastic lesions (Zou et al., 2004). In a mouse breast cancer model, overexpression of cyclooxygenase 2 (COX2) resulted in an increased recruitment of Tregs to the tumor and increased bone metastasis indicating the role of Tregs in promoting colonization and proliferation of breast cancer cells to the bone. This was further substantiated by decreased bone metastasis of breast cancer (Huppert et al., 2022) cells upon inhibition of CXCL12/CXCR4 axis and indoleamine 2,3-dioxygenase (IDO1) expression (Huppert et al., 2022).

Furthermore, it is anticipated that the bone microenvironment fosters a pro-metastatic niche feedback loop as under metastatic conditions (Huppert et al., 2022), recruitment of Tregs to the BM is increased via the action of the CXCR4/CXCL12 signaling pathway. Within the bone, RANK ^+^ DCs induce Treg cell expansion, which themselves serve as the key source for RANKL production in the presence of tumors, further inducing bone resorption that leads to local acidification of the bone microenvironment thereby activating TGF-β and generating more Tregs which promote tumor cell proliferation and growth within the bones (Huppert et al., 2022).

MDSCs owing to their remarkable immunosuppressive capabilities, which include the inhibition of T cell proliferation and antigen-specific T cell responses and decreased cytokine production intricately contribute to cancer progression including secondary bone metastasis (Li et al., 2024a). However, their role in breast cancer-associated osteolysis and bone metastasis extends beyond immunosuppression, as evident by increased MDSCs expansion and bone destruction in intraorthotopic and intracardiac breast cancer models of nude mice (Danilin et al., 2012). It has been hypothesized that MDSCs serve as the growth and invasion factors creating a locally favorable microenvironment in the bone to promote tumor growth (Danilin et al., 2012). Notably, in tumor-bearing mice, MDSCs in bone marrow exhibit increased expression of TGF-β1 that stimulates breast cancer cells to express certain factors e.g., parathyroid hormone-related protein (PTHrP) and GLI2 that are essential for breast cancer-induced osteolysis. Furthermore, a vicious cycle leads to MDSC expansion during breast cancer and increased dissemination of the breast cancer cells to the bone (Liu et al., 2023a). The tumor-derived factors including macrophage colony-stimulating factor (MCSF), COX-2, interleukins like IL-6, 13, 17 promote the expansion of MDSCs exhibiting increased expression of TGF-β (Liu et al., 2023a). In addition, MDSCs promote tumor angiogenesis via increased MMP9 secretion which further increases VEGF bioavailability, ultimately leading to TFG-β overexpression which is associated with bone destruction (Han et al., 2024). Besides, there is compelling anticipation that MDSCs possess the capacity to differentiate into cell types deemed essential for tumor growth. This proposition was substantiated by the observed ability of Gr1^+^CD11b^+^ cells to differentiate into osteoclast precursors both in vitro and in-vivo. Notably, in orthotopic mice models, the augmentation of MDSCs expansion was positively correlated with an increased population of OC precursors (Danilin et al., 2012; Sawant et al., 2013).

Bone-marrow-associated macrophages (BMMs) derived from the classical monocytes promote breast cancer metastasis to bones in an IL4R^+^ dependent manner which is anticipated to be crucial for the polarization and pro-tumor function of BMMs (Chen et al., 2024; Ma et al., 2020b). As a point of evidence, mosaic mice bearing IL4ra^−/−^ exhibited decreased bone colonization of breast cancer cells compared with IL4ra^+/−^ mosaic mice. It is suggested that IL4R regulates tumor-promoting functions rather than BMMs recruitment as substantiated by the comparable abundance of monocytes and macrophages in both the mice groups. The comparable abundance of osteoclasts in mosaic mice suggested the significance of IL4R exclusively in promoting BMMs-induced bone metastasis with no direct effect on bone remodeling (Ma et al., 2020b). Moreover, it has been elucidated that macrophages derived from inflammatory monocytes rather than the BMMs are responsible for bone metastasis. This assertion was corroborated by a notable reduction in bone metastatic growth in mice deficient in chemokine (C-C motif) ligand-2 (CCL2), an important factor responsible for the recruitment of inflammatory monocytes to the bone (Ma et al., 2020b). In contrast, no such effects were observed upon ablation of bone marrow resident macrophages via diphtheria toxin (DT) treatment in transgenic mice expressing the DT receptor gene under the control of CD169 promoter- a cell surface marker specific to BMMs (Ma et al., 2020b).

## 4 Gut microbiome and bone homeostasis

**Fig. 3:**
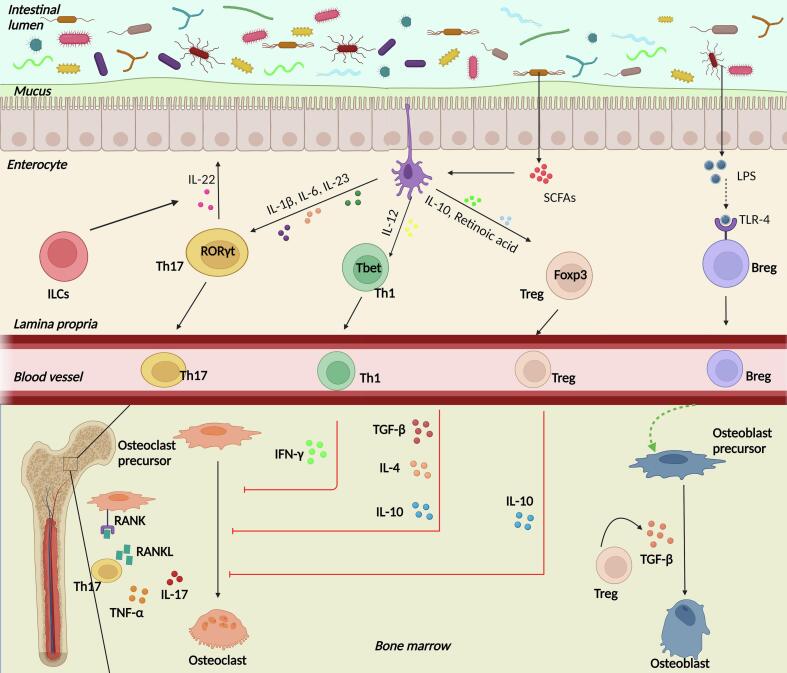
**The nexus between gut microbiota (GM) and bone**. The GM maintains bone homeostasis and under conditions of dysbiosis, results in abnormal bone remodeling contributing towards osteo-pathologies. A leaky gut allows the translocation of GM species from the intestinal lumen into the lamina propria which hosts a multitude of immune cells. GM species and derived short chain fatty acids (SCFAs) induce the dendritic cells (DCs) to stimulate Th17 and Th1 cells which further participate in inhibiting osteoclastogenesis via the production of cytokines such as interferon (IFN)-γ, interleukin (IL)-10, IL-4, and transforming growth factor (TGF)-β. Secretion of a variety of interleukins drive activation of Th17 cells which promote osteoclastogenesis via tumor necrosis factor (TNF)-α, IL-17, and receptor activator for nuclear factor kappa-B (RANK)- receptor activator for nuclear factor kappa-B ligand (RANKL) signaling. Also, production of IL-22 by Th17 and innate lymphoid cells (ILCs) maintains gut membrane integrity. Bregs are activated directly by the bacteria-derived lipopolysaccharide (LPS) via toll like receptor-4 (TLR4) signaling which inhibits maturation of osteoclast precursors directly and indirectly by inducing Tregs to suppress bone resorption. Secretion of TGF-β by Tregs function to convert pre-osteoblasts into osteoblasts, thus inducing bone formation.

The colonization of GM begins at birth when the neonate is exposed to vaginal and environmental microbial flora. GM changes with age and exhibits high variability in elderly individuals compared to younger adults (Claesson et al., 2011). Recently, osteomicrobiology has emerged as a novel interdisciplinary field that dictates the role of GM in bone homeostasis, combining bone physiology, gastroenterology, immunology, and microbiology. The human GM collectively refers to the trillions of microorganisms diversifying from acellular to cellular prokaryotes (bacteria and archaea) and eukaryotic microbes that inhabit the gastrointestinal tract (Shreiner et al., 2015). The first evidence of the association between GM and bone was elucidated in a study by Sjögren et al. where they found a significantly higher trabecular bone volume and reduced level of osteoclastogenesis in bones of germ-free (GF) mice as compared to that of conventionally raised (ConvR) mice (Sjögren et al., 2012). Dysregulated bone remodeling because of GM dysbiosis has been observed in patients with bone pathologies. Based on the results of 16S rRNA gene sequencing in patients with osteoporosis and primary osteopenia, it was hypothesized that the immune-inflammatory axis act as a bridge between GM and bone metabolism (Wang et al., 2017). This may be attributed to altered levels of insulin growth factor (IGF)-1, tumor necrosis factor (TNF)-α, and IL-1β or alterations in relative abundances of osteoclasts, osteoclast precursor cells, CD4^+^ T cells, and inflammatory cytokines (Fig. 3) (Yan et al., 2016).

On the contrary, short-chain fatty acids (SCFAs) derived from the GM exert anti-inflammatory effects in the intestinal mucosa by histone deacetylases (HDACs) inhibition and G-protein-coupled receptors (GPCR) activation in IECs. This in turn affects gene regulation of cell proliferation, differentiation, and inflammatory response thus protecting from diseases.

A study by Lucas et al. revealed propionate and butyrate-induced metabolic reprogramming of osteoclasts and subsequent downregulation of essential osteoclast genes- TNF receptor-associated factor 6 (TRAF6) and nuclear factor of activated T cells (NFATc1), thus acting as potent regulators of bone homeostasis (Lucas et al., 2018). In the case of microbial dysbiosis, altered immune responses lead to increased bacterial translocation from the intestinal lumen into the lamina propria accompanied by the release of pro-inflammatory cytokines (Lyu et al., 2023). A prolonged state of inflammation often results in chronic inflammatory diseases that are associated with bone destruction, such as inflammatory bowel disease (IBD). Certain SCFAs e.g., acetate assist in ameliorating ovariectomy (ovx)-induced bone loss by decreasing the number of osteoclasts in a T-cell and B-cell dependent manner. A study identified that nucleotide-binding oligomerization domain (NOD1) and NOD2 signaling affects bone mass in response to external stimuli, like GM. Bacterial ligand stimulation of NODs resulted in osteoclastogenesis via increased RANKL and TNF-α in the bone of ConvR mice (Ohlsson and Sjögren, 2018). From such studies, it is derived that colonization with GM leads to activation of the immune system, especially CD4^+^ T cells which directs osteoclastogenesis via increased levels of pro-inflammatory cytokines in bone (Ohlsson et al., 2017).

### 4.1 GM Dysbiosis and bone metastases

**Fig. 4:**
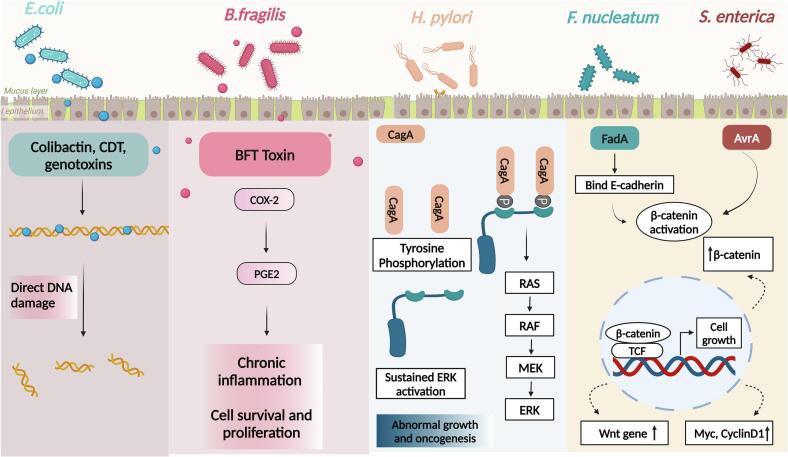
Microbial inducers of carcinogenesis. Genotoxin-producing bacteria *E. coli* and *B. fragilis* drive carcinogenesis by inducing direct DNA damage and cyclooxygenase (COX)-2 mediated signaling, respectively. *H. pylori* produces cytotoxin-associated gene A (CagA) bacterial protein which bind to the phosphorylated Src homology 2 (SH2) domain leading to an increase in ERK levels driving oncogenesis. The virulence factors produced by *F. nucleatum* and *S.enterica* induce activation of β-catenin which upon translocation to the nucleus results in overexpression of a variety of genes involved in cell proliferation, such as Myc, CycinD1, Wnt etc.

GM has been recognized to play a crucial role in cancer progression (Balkwill and Mantovani, 2001; Lu et al., 2014; Tjalsma et al., 2012). Primarily, certain microorganisms such as *Escherichia coli* and *Shigella dysenteriae* are known to secrete DNA-damaging toxins e.g., colibactin and cytolethal distending toxin (CDT), which are known to participate in chronic inflammation and tumor progression (Guerra et al., 2011). Additionally, microbes secrete certain pro-tumorigenic metabolites e.g., lithocholic acid (LCA) which have a role in promoting colorectal cancer in an IL-8-dependent manner (Fig. 4) (Ivleva and Grivennikov, 2022).

To begin with, altered GM profiles in osteosarcoma mice models provide a proof of concept for establishing a cross talk between the gut microbiome and bone malignancy. The OS mouse models exhibited higher alpha diversity than controls, characterized by a significant decrease in the relative abundance of *Lachnospiraceae* spp. and an increased Firmicutes/Bacteroidetes (F/B) ratio (Le et al., 2023). This shift was largely driven by a decrease in the *Bacteroides* spp. and a simultaneous increase in *Firmicutes* specifically *Roseburia* spp. and *Akkermansia*. The increased *Roseburia* population likely triggers butyrate production in the gut subsequently enhancing mucin production by colonocytes which upon degradation by *Akkermansia* spp. enhances the alpha diversity thereby establishing a favorable gut environment as a response to this malignant condition. This corroborates with previous findings showing a considerable association of high alpha diversity with patients' response to chemotherapy. In melanoma mouse models, the GM has been recognized to play a pivotal role in restraining the progression of tumor-induced bone metastasis, as it regulates the immune cells' trafficking to the bone. To investigate the role of the GM, the resident microbes were ablated to mimic dysbiosis using broad-spectrum antibiotics, and B16-F10 melanoma cells were administered. Antibiotic induced-GM depletion significantly enhanced intraosseous tumor growth and osteolysis as revealed by increased tumor burden and decreased perforation number, volume, thickness, and ectopic bone volume (BV) compared to mice with intact GM. This finding was in corroboration with earlier literature demonstrating enhanced breast cancer growth upon antibiotic manipulation of the GM in mice (Le et al., 2023).

Furthermore, the antibiotic-induced GM depletion also prevented intestinal egress of anti-tumorigenic natural killer (NK and type 1 T helper (Th1)) cells to the bone marrow. In the case of breast cancer, the GM analysis revealed that certain microbial species were particularly abundant in patients with breast cancer bone metastasis such as *Streptococcus*, *Campylobacter*, *Moraxellaceae*, etc. These patients also lack the presence of certain beneficial bacterial species (*Megamonas* and *Akkermansia*) (Wenhui et al., 2022). Another shotgun metagenomic analysis by Zhu and colleagues shows the relationship between GM dysbiosis and breast cancer development in post-menopausal patients, in which it was observed that *E. coli*, *Klebsiella*, *Prevotella amnii*, *Enterococcus gallinarum*, *Actinomyces* sp., etc. were enriched in post-menopausal-breast cancer (PMOB) patients (Zhu et al., 2018). Additionally, a mouse model study showed that administering antibiotics led to faster tumor growth that is anticipated to be caused due to mast cell homing and/or function (McKee et al., 2021). Breast cancer commonly metastasizes to the bone as mentioned previously, which has a rich environment of calcium and minerals. It can thus be hypothesized that GM dysbiosis leads to breast cancer development which is eventually metastasized to bones.

16S rRNA sequencing reveals gut dysbiosis in prostate cancer patients leading to tumor progression and its metastasis. It is observed that *Ruminococcus* species is extensively upregulated in human clinical samples as well as mouse models of prostate cancer (Liu et al., 2021). The Fecal microbial transplantation of castration-resistant prostate cancer mice feces into TRAMP (Transgenic adenocarcinoma of the mouse prostate) mice resulted in an enhanced tumor progression. The gut microbiota dysbiosis is associated with prostate cancer progression potentially by the “LFCAT1-DNA repair pathway” (Liu et al., 2021). The bacterial sps i.e. *Ruminococcus*, *Alistipes*, and *Phascolarctobacterium* also release certain GAMs (Gut-associated metabolites) such as Short-chain Fatty Acids (SCFAs) like acetate and butyrate that regulate prostate cancer progression by many pathways. These SCFAs induce autophagy in cancer cells followed by NF-kB and MAP-kinase pathway activation along with macrophages subsequently promoting tumor growth (Liu et al., 2023b). Androgen deprivation in mice and humans has been found to promote expansion of defined commensal microbiota triggering the onset of castration resistant prostate cancer (Terrisse et al., 2022). These findings suggest that either extraneous supplementation of androgen or use of some GM modulation strategies to increase the number of androgen synthesizing flora may contribute to bone metastases.

In the aforementioned studies, the common thread of gut dysbiosis alongside its association with bone metastasis either directly or via immune modulation, implies a potential mechanistic link between the two. In forthcoming sections, we will discuss specific immune components governing bone metastasis that can be targeted via microbiological interventions, further establishing an interplay between GM and bone metastasis.

### 4.2 Intratumoral microbiome and metastases

Intratumoral microbiome (ITM), characterized by its dynamic and diverse composition, has recently been recognized as an integral and intrinsic component of the tumor tissue instead of an incidental presence. The tumor microenvironment owing to the hypoxic, nutrient-enriched, and immunosuppressive conditions, provides a favorable niche for microbial colonization (Lu et al., 2024; Yang et al., 2023). Destructed mucosal barriers, dysbiotic gut, adjacent tissue migration, and hematogenic invasion are considered to be the sources of microorganisms in tumor tissues wherein these organisms either directly or indirectly influence cancer initiation and progression or modulate the immune response by different signaling pathways (Cao et al., 2024; Yang et al., 2023; Yousefi et al., 2024).

ITM directly or via secreted metabolites affects epigenetic modifications that aberrantly activate oncogenes or suppress tumor suppressor genes thereby affecting cancer progression (Wu et al., 2024). For instance, SCFAs modulate the activity of histone acetylase and deacetylase, subsequently triggering genomic epigenetic changes. As a point of evidence, a study by Ma et al. suggested that butyrate promotes lung cancer metastasis and decreases the likelihood of recurrence-free survival in patients. This was evidenced by a higher abundance of butyrate and butyrate-producing microbiota e.g., *Roseburia* in the tumor tissues of individuals with reoccurring lung cancer than the non-recurring group. Microbiome-derived butyrate, when present in low concentration in the tumor site, inhibited the expression of HDAC, a common deacetylating agent. This inhibition promoted histone H3K27 acetylation at the H19 promoter which upon activation instigates downstream factors viz., MMP15, pivotal in regulating tumor metastasis and progression. Additionally, butyrate promoted secretion of the various cytokines notably IL-10 and IL-13, thereby triggering polarization of M2 macrophages. This facilitates immune evasion, promotes angiogenesis, and remodels local tissue, consequently establishing a tumorigenic milieu. Intriguingly, this metabolite elicited an upregulation in the expression of genes crucially implicated in cell adhesion and connection e.g., neuroactive ligand-receptor interaction and extracellular matrix interaction thereby promoting metastasis (Ma et al., 2024). Conclusively, this study provides a cross-talk between intratumor microbiome and cancer metastasis. Along similar lines, *L johnsonii* in collaboration with *C. sporogenes* converts dietary tryptophan to Indole-3-propionic acid that promotes the infiltration of CD8^+^ T cells into tumor sites and enhances the H3K27 acetylation in the Tcf7 gene thereby modulating the stemness of these cells and facilitating the generation of progenitor exhausted CD8^+^ T cells (Tpex) subsequently ameliorating the efficacy of anti-PD-1 immunotherapy (Jia et al., 2024b). The above two studies indicate the potential of intratumoral microbiota-derived metabolites to be utilized as drug adjuvants for patients receiving personalized cancer immunotherapy.

Besides, ITM interacts with the immune system and can foster an immunosuppressive microenvironment and immune cell inactivation. *F. nucleatum* colonization in breast cancer tissues, facilitated by high Gal-GalNAc levels on breast cancer cells, supports metastasis by suppressing the accumulation of tumor-infiltrating T cells (Parhi et al., 2020). Alternatively, ITM can interact with pattern recognition receptors in the tumor microenvironment thereby activating inflammatory pathways and cascade. The circulating tumor cells are characterized by the presence of microorganisms suggesting the ability of ITM together with the host tumor cells; to travel through the circulation system to the distal organ wherein these can alter internal features of oncocytes and the external conditions to establish a microenvironment conducive to metastasis. With the progression of metastatic growth, the microbiota is influenced by the distal organ environment, which explains the different ITM profiles in the primary tumor tissue and the site of metastasis. Moreover, ITM can modulate the cellular cytoskeletal system and significantly relieve the mechanical stress-induced contractile forces on the tumor cells thereby enhancing tumor cells' viability during metastasis. As a point of evidence, *Staphylococcus*, *Lactobacillus*, and *Streptococcus* residing in breast cancer tissues are known for restraining the RhoA/ROCK signaling pathway to remodel the actin skeleton, increasing tumor cells' resistance to fluid shear stress that is commonly experienced by circulating cells after intravasation responsible for their apoptosis (Fu et al., 2022). A similar observation has been made with respect to colorectal cancer and could hold for other cancer types as well. Certain microorganisms can promote cancer cell adhesion to endothelial cells by triggering NF-κB signaling pathway and upregulating intercellular adhesion molecule (ICAM)-1. Recent studies have unveiled a positive association between tumor microbial diversity and infiltrating neutrophils and macrophages suggesting that bacterial colonization within the tumor tissues contributes to an immunosuppressive environment by establishing neutrophil extracellular traps (NET)-based shielding of tumor cells from cytotoxic killing (Battaglia et al., 2024; Blake et al., 2024).

**Fig. 5:**
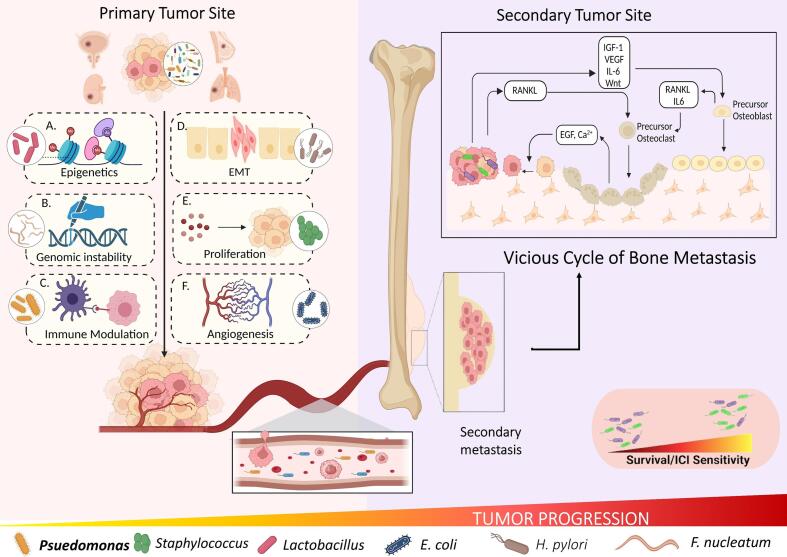
Intratumoral microbiota governing cancer progression: Microbiota residing within the tumor tissues affect the cancer progression by several mechanisms A. Epigenetic modifications involving histone acetylation; B. Genetic modification; C. Immunomodulation; D. Epithelial to Mesenchymal transition*;* E. Increased cancer cell proliferation; F. Increased Angiogenesis*.* Either of these mechanisms individually or collectively, allows the tumor cells to metastasize which is usually to bone in case of Breast, Prostate, Lung and Renal cancer. Within the bone, microbiome along with the tumor cells travel to the site of secondary tumor and create microenvironment conducive for metastasis. The translocated microbiota in conjunction with the resident microbiota of that tissue decide the survival of the patient and their response to immune checkpoint inhibitors (ICI) therapy.

Furthermore, ITM-derived metabolites have a significant impact on tumor immunity and response to immune checkpoint inhibitors (ICI) therapy (Table 2). As a point of evidence, the presence of *Clostridiales* derived trimethylamine N-oxide in the breast cancer tissues triggered gasdermin E mediated pyroptosis in tumor cells via activation of endoplasmic reticulum stress kinase PERK (Wang et al., 2022a). Another in-vivo experiment unveiled that *Lactobacillus reutri* (LR) colonizes the tumor microenvironment and via secretion of an immunomodulatory, tryptophan catabolite i.e., indole-3-aldehyde drives spontaneous antitumor immunity and potentiates ICI therapy (Bender et al., 2023b). I3A being an aryl hydrocarbon receptor (AhR) agonist, directly acts upon CD8^+^ T cells and promotes IFN-γ production. It was reported that I3A-induced immunity was constrained to the tumor microenvironment, thereby suggesting the importance of the local environment in governing the sensitivity of tumor-infiltrating CD8^+^ T cells to type 1 CD8^+^ T cells (Tc1) cell-promoting factors (I3A in this case). Additionally, LR treatment elevated the recruitment of CD8^+^ T cells to the tumor site by promoting the expression of Ccl5 and Ccl4 genes. I3A simultaneously increased the cytotoxic granzyme B production in the recruited CD8^+^ T cells via the upregulation of essential transcription factors *Blimp1* and *lfng*. Combinatorial treatment including LR along with anti-PD-L1 or anti-CTLA-4 therapy exhibited pronounced cytotoxic T-cell response in the tumor (Bender et al., 2023b) microenvironment and better control than the ICI therapies given individually. Lastly, I3A was found in higher abundance in the ICI responder melanoma patients thus indicating that the metabolites derived from intratumoral microbiota have the potential to serve as biomarkers for predicting ICI sensitivity (Fig. 5) (Bender et al., 2023b).

**Table 2:** The ability of microbial species to modulate anti-tumor responses and bone homeostasis.

Microorganism	Cancer targeted	Impact on ICI therapy/anti-tumor response		Additional role in bone health/homeostasis^a^	References
		Immunotherapy given	MOA of ICI promotion/anti-tumor activity		
*Bifidobacteria*	Breast	Anti-PD-1 therapy	Increased NK cell tumor infiltration, decreased production of pro-tumor macrophages	Suppresses differentiation and functional activity of RANKL-induced osteoclaststs	(Di Modica et al., 2021; Kim et al., 2021; Sapra et al., 2022)
*Lactobacillus*	Breast	anti-PD-1/PD-L1	Indole acetic acid production and CD8^+^ T cell activation via AhR signaling	Improve osteogenesis and inhibit osteoclastogenesis	(Peng et al., 2020; Sapra et al., 2021)
*Prevotella*	Prostate	–	Decrease levels of androgens and delayed onset of cancer	Inhibits osteoclastic bone resorption and slows down bone loss in PMO	(Chen et al., 2023; Fujita et al., 2023; Lee et al., 2014; Pernigoni et al., 2021; Ponzetti and Rucci, 2019)
*Akkermansia*	Lung cancer	–	Enhanced CD8^+^ cytotoxicity, granzyme and IFN production, supressed PDL-1 expression	Increase in osteoblast population and improves bone quality	(Keshavarz Azizi Raftar et al., 2021; Xu et al., 2024)

PD-1: Programmed Cell Death Protein 1, PD-L1: Programmed Cell Death Ligand 1, DC: Dendritic cell, AhR: Aryl hydrocarbon receptor, IL: Interleukin, pTregs: Peripherally derived Tregs, IFN: Interferon, MDSCs: Myeloid-derived suppressor cells, RANK-L: Receptor activator for nuclear factor kappa-B ligand, PMO: Post-menopausal osteoporosis.

a This column is unrelated from the cancer-studies mentioned in rest of the columns of the table but instead it includes independent studies demonstrating the contributory role of corresponding bacterial species in maintaining bone homeostasis. Thereby suggesting the prospect of using these bacterial species for managing the metastasis of the primary malignancies like breast and prostate to the bone.

While the aforementioned studies may not directly align with bone metastasis, we took into consideration the malignancies with a higher likelihood of metastasizing to bone as the former is still unexplored. This broader perspective will facilitate the extrapolation of relevant principles and methodologies for better understanding and management of bone metastasis. By examining analogous scenarios and identifying how the Intratumoral microbiome interacts with the osteo-immune system, we stand to deepen our understanding of the disease progression and concurrently design a robust framework for future investigations aimed at identifying novel therapeutic targets and strategies.

## 5 Targeting “microbiome-immune-bone axis” in metastases

The conventional treatment for primary bone cancer involves surgical resection followed by chemotherapy and radiotherapy, but these treatments have limited efficacy against secondary bone cancers like those from the breast and prostate (Ferguson and Turner, 2018). Currently, anti-resorptive agents like bisphosphonates and denosumab are used to prevent skeletal-related events (SREs) in patients with bone metastases (Hofbauer et al., 2021). Despite advancements, managing bone metastases remains challenging. However, recent research suggests the potential of GM in improving the cancer treatment outcomes. Clinical and pre-clinical studies provide strong evidence that modulating the gut microbiome to reverse the established microbial dysbiosis can improve patient outcomes. This can be achieved by developing novel therapeutic strategies including fecal dietary interventions such as prebiotics probiotics, etc. (Knisely et al., 2023).

**Probiotics**, like *Lactobacillus* species have shown anticancer effects in murine breast cancer model by immune response modulation via cytokines viz., IFNγ, TGF-β etc., (Maroof et al., 2012). Besides, *Lactobacillus rhamnosus* GG (LGG) supplementation in sex-steroid deficient female mice improved the intestinal permeability and simultaneously reduced the levels of osteoclastogenic cytokines (Li et al., 2016a). *Lactobacillus reutri* have been shown to promote anti-tumor immunity in breast cancer by promoting interferon-γ-producing CD8^+^T cells and its derived tryptophan metabolite was demonstrated to enhance ICI therapy (Bender et al., 2023a, Bender et al., 2023b). Taken together, these studies present the prospect of extrapolating these benefits of probiotics for managing bone metastasis.

**Prebiotics**, such as oligofructose and inulin, promote beneficial bacteria growth while reducing inflammation and cell proliferation linked to colorectal cancer. Rivera-Huerta et al. demonstrated that inulin-type fructans (ITF), derived from chicory and Mexican blue agave has a significant effect on bone metrics and gut health. Their findings unveiled that ITF supplementation enhanced calcium absorption, which holds significant implications for bone health. Furthermore, following ITF therapy, examinations of bone densitometry showed a noteworthy improvement, indicating possible advantages for bone strength and density. The significant reduction in TNFα levels, a key proinflammatory cytokine, coupled with elevated IL-10 levels following ITF therapy, underscores its potential in cancer treatment. Given the intricate interplay between inflammation and cancer progression, the anti-inflammatory properties of ITF could extend to bone metastasis management (Rivera-Huerta et al., 2017).

**Postbiotics**, derived from beneficial gut bacteria, have shown potential in cancer therapy. Compounds like butyrate and tributyrin induce tumor cell apoptosis, while hydrogen sulphide provides cytoprotective effects. SCFAs, produced by gram-positive, gut-resident bacteria are presumed to mediate bone homeostasis by restoring IGF-1 levels (Yan et al., 2016). IGF-1 has been hypothesized to play a crucial role in mediating DTCs' interaction with the bone microenvironment in various types of cancers e.g., neuroblastoma, breast cancer and prostate cancer (Rieunier et al., 2019). This hypothesis is supported by decreased bone metastases in mice BC models transfected with 486STOP (dominant negative IGF-1R). Similarly, non-obese diabetic-severe combined immunodeficient (NOD-SCID) mice implanted with human adult bone and injected with MDA PCa 2b cells, exhibited decreased PCa induced bone metastases upon treatment with anti-IGF-1R antibody (Goya et al., 2004). Conclusively, IGF-1R appears as a promising target for developing therapeutic interventions for managing bone metastasis.

**Fig. 6:**
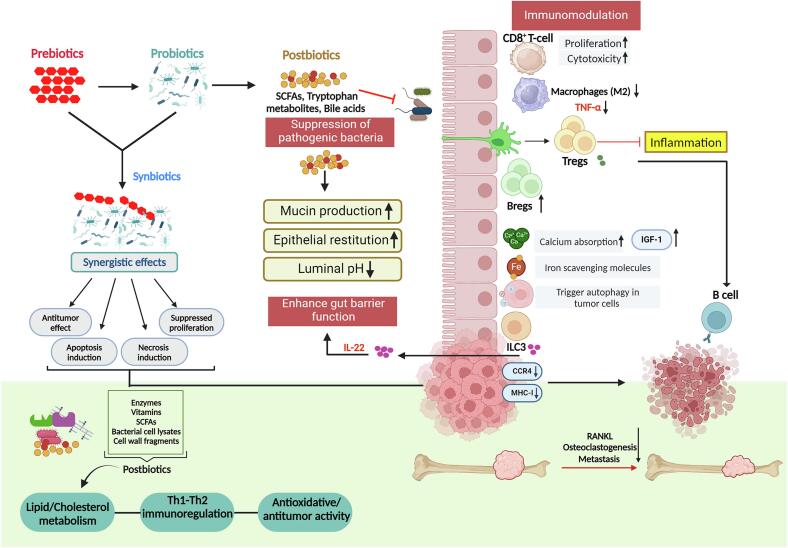
Modulation of gut microbiota (GM) for therapeutic purposes. Prebiotics and probiotics, including the gut-associated short-chain fatty acids (SCFAs) exert anti-cancer effects by preventing the epithelial breach by pathogenic bacteria. This is mediated by activation of the immune machinery to enhance the integrity of gut barrier function by release of interleukin (IL)-22 from innate lymphoid cell (ILC)3s. Activation of dendritic cells (DC)s stimulate Th17 cells to produce IL-17A which has an anti-tumor effect. SCFAs also play a crucial role in maintaining luminal pH and mucin secretions, keeping up with the epithelial integrity. Synbiotics and postbiotics, which include bacterial cell lysates, enzymes, vitamins, SCFAs, and cell wall fragments, also have anti-cancer effects resulting in tumor regression.

Altogether, the above-mentioned studies, alongside the existing literature on microbial interventions in cancer metastasis (Table 3 & Fig. 6), lay a compelling foundation suggesting the commendable role of microbiota in modulating immune cells' behaviour or influencing bone cells involved in bone remodeling.

**Table 3:** Microbiological-based interventions in treating various cancer types.

Nutritional supplement	Example	Cancer type targeted	Mode of action	Reference
Probiotics	*Lactobacillus* species	Murine breast cancer model	Activate NK cells.	(Aragón et al., 2015; Hibberd et al., 2017; Li et al., 2016b; Sapra et al., 2021, Sapra et al., 2022; Zitvogel et al., 2017)
			Promote dendritic cell maturation.	
			Release iron-scavenging molecules.	
	*Lactobacillus acidophilus* and *Bifidobacterium animalis*	Colon Tumors	Modify the microbiota favoring butyrate-producing bacteria.	
	*Bifidobacterium longum*	Colorectal Cancer	Butyrate in turn, inhibits cell growth, induces apoptosis, and diminishes inflammation	
	*Lactobacillus rhamnosus GG (LGG)*	Colorectal Cancer	Suppresses RANKL-induced osteoclastogenesis	
			Enhances gut permeability.	
			Reduces Osteoclastogenic cytokines	
Prebiotics	Non-digestible polysaccharides	Ulcerative colitis patients with a risk of colorectal cancer	Increase SCFAs and SCFA receptor expression, reducing inflammation and cell proliferation.	(Hu et al., 2016; Li et al., 2020; Taper and Roberfroid, 2000; Zitvogel et al., 2017)
	Oligofructose or Inulin	Breast, Colon and Lung Cancer	Enhance the curative properties of many cytotoxic medications, thus improving chemotherapy effectiveness.	
	Mucin or inulin polysaccharides	Melanoma mice models	Alter tumor microenvironment leading to tumor ablation.	
Synbiotics	*Lactobacillus casei* spp. along with dextran	Colorectal Cancer	Increased NK cell activity.	(Ogawa et al., 2005; Rafter et al., 2007; Saito et al., 2019)
			Lower production of inflammation mediators like COX-2, STAT3, IL-6.	
			Decreased cell proliferation and improved mucosal structure.	
Postbiotics	Tributyrin		Trigger autophagy, preventing tumor cells from metastasizing.	(Vrzáčková et al., 2021)
	Indole-3-Lactic acid	Colorectal Cancer	epigenetically induced increased IL12a production in DCs, subsequently enhancing CD8^+^ T cell function	
	H_2_S		Causes Tissue damage at very high concentrations yet at low level, acts as a cytoprotective agent.	

NK: Natural Killer, RANK-L: Receptor activator for nuclear factor kappa-B ligand, SCFA: Short chain fatty acids, COX-2: Cyclooxygenase-2, STAT3: Signal transducer and activator of transcription 3, IL: Interleukin, DC: Dendritic cells.

## 6 Challenges and future perspectives

The above-mentioned studies highlight a striking variability in effects observed with microorganisms and their metabolites, which intricately depends upon the specific bacterial strain and cancer type taken into consideration. Notably, butyrate, recently recognized for its role in maintaining bone health, serves as a prime example of this variability. While it markedly attenuated prostate cancer progression by inducing autophagy, its presence within the lung tumor tissues paradoxically facilitated metastatic dissemination via epigenetic modifications, both of which have a substantial risk of bone invasion. Furthermore, it has been observed that at low concentrations, butyrate significantly promoted cancer cell proliferation, whereas at high concentrations, it unexpectedly suppressed cell proliferation (Oncel et al., 2024). This divergence underscores a complex interplay between microbial-derived metabolites, their localization, cancer type, and their progression. Furthermore, the bacterial composition and richness of different cancer types are highly heterogeneous. Given this heterogeneity of microbiota and their divergent roles, it is crucial to acknowledge that the metabolites' effects are highly context-dependent, and benefits observed in one type of malignancy may not seamlessly extrapolate to another. Though recent studies have started to recognize the presence of microorganisms in tumor sites as well, the gut still stands to be the biggest reservoir for colonising microorganisms with access to all the other organs of the body. Thus, there is a high possibility that the GM and immune system may interact with intratumor microbiota to determine cancer progression. Drawing from these points, it can be concluded that the effectiveness of microbiota-focused treatments significantly depends on the interplay between the functioning of the gut and tumor-resident microbiota with the immune system and their influence on the local and systemic environment. Investigating these facets concurrently would unveil novel approaches for developing prevention and treatment modalities to manage bone metastasis. The ideal course of action is to investigate microbiota-based techniques targeting both gut and tumor microbes in larger patient groups (Wang et al., 2023).

An in-depth understanding of the interplay between intestinal and intratumor microbiomes in conjunction with the immune system holds the promise to stratify patients into responders and non-responders. Given the heterogeneity of the microbiome, this approach will allow for the development of personalized therapeutic interventions tailored to individual microbiome profiles. Microbiological-based interventions, including probiotics, prebiotics, and postbiotics, will assist in altering gut or intra-tumor microbiota for reverting non-responder patients to a responsive state and enhancing the efficacy of administered therapies. Considering all these aspects, we propose leveraging microbiota-based interventions to reconfigure the immune environment in bone tissue. However, the microbiomes, being complex ecosystems, are rapidly evolving, and so are the tumor cells and the tumor microenvironment. Hence, it is difficult to effectively comprehend the intricate relationship between tumor and GM in the context of cancer progression. The efforts are further complicated by the accessibility constraints associated with low biomass microbial components and ethical challenges associated with handling human tissues. The utilization of interdisciplinary strategies involving bioinformatics and multi-omics approach such as metagenomics, meta-transcriptomics, and metabolomics, will foster a deeper understanding of the mechanisms underlying microbiome-tumor interactions. In conjunction with computational approaches for high-dimensional analyses of the established bacterial genera in the tumor microenvironment on a large scale, advanced techniques involving multiple integrated imaging and analysis methods, spatial and cellular localization of the tumor-resident microorganisms will deepen our comprehension of host-microbiome interactions (Battaglia et al., 2024; Cao et al., 2024; Fu et al., 2022). The incorporation of synthetic biology techniques will enable the rational engineering of microorganisms for the prevention and treatment of metastasis. Furthermore, artificial intelligence and machine learning approaches can be explored for designing models trained with large, multidimensional datasets obtained from the muti-omics and sequencing studies. This will streamline the patient stratification based on state-specific microbial signatures, thereby precisely and non-invasively predicting the patients' sensitivity to immunotherapy. Furthermore, machine learning platforms will assist in the expansion of public repositories that will provide more human microbiome-omics data and enable researchers to identify new associations between the microbiome and malignancy (Novielli et al., 2024). Owing to its ability to provide, high-confidence taxonomic and functional characterization of tumor and gut resident bacteria, this approach will bring about a fundamental paradigm shift in the field of oncology from observational association studies to experimental causal inference and clinical intervention (Battaglia et al., 2024; Li et al., 2022). It is anticipated that machine learning and artificial intelligence will instigate the development of microbe-based or microbe-directed clinical interventions, especially when used in combination with emerging technologies such as cultivation-free genome sequencing and the manipulation of gut and tumor microbial genes. We believe that this approach holds great promise for the future of immune-onco-microbiome research (Giuffrè et al., 2023). With the standardization of protocols, development of robust sequence analysis strategies, and development of reliable databases, these strategies hold the potential to revolutionize the management of bone metastatic cancer. This promotes an immunostimulatory landscape while strategically regulating the bone remodeling process. Diverging from conventional research paradigms that often focus on singular aspects, this novel approach utilizing microorganisms concurrently targets immune modulation and bone remodeling, ultimately maintaining skeletal stability amidst metastatic cancers. The convergence of these scientific shreds of evidence and clinical insights represents an opportunity for a paradigm shift in managing bone metastasis. Exploring combination therapy strategies based on microbial interventions to improve the clinical effect will provide promising research direction.

## 7 Conclusion

In a nutshell, the increased propensity of bone metastasis in breast and prostate cancer patients with dysbiotic gut, alongside altered GM profiles in osteosarcoma mouse models and increased bone metastasis in melanoma mice models with depleted GM, shed light on the intriguing interplay between gut microbiota and bone metastasis. Dysbiotic gut could potentially foster an environment conducive to the proliferation and survival of metastatic tumor cells within the bone via multiple mechanisms e.g., regulation of critical immunological components including IGF-1 levels, IL-6, Tregs, etc. Besides, tumor tissues harbor distinct microbial communities that are either the inherent residents of that organ or originate from different sources e.g., disrupted mucosal barriers. As an integral part of tumor tissues, these microorganisms significantly affect cancer physiology via various mechanisms involving epigenetic modifications, immune evasion, etc. Not only do they modulate cancer progression, but also have the potential to influence patients' sensitivity to immunotherapies. Since majority of the crucial pathways are closely linked to the immune system components, in the crosstalk between the human microbiome and metastatic environment, one thing that stays constant is the osteoimmune framework linking bone metastases and microbiome. Identifying this convoluted connection will provide important information about possible treatments for bone metastases. The integration of microbial interventions with immunotherapy or chemotherapy has the potential to navigate drug resistance and augment anticancer efficacy. The existing studies offer a theoretical framework that requires the utilization of similar approaches to identify the individual bacterial mediators and metabolites involved in osteo-carcinogenesis and delineate their interaction with osteometabolites and osteoimmune systems to subsequently decipher the cumulative role of gut and tumor microbiota on bone metastasis. This field has immense flourishing potential yet interdisciplinary comprehensive approaches are required for further exploration. Transformative therapeutic modalities can be established by fostering synergy across diverse fields which can revolutionize the landscape of bone metastasis.

## List of abbreviations

ICI: immune check Inhibition
NET: neutrophil extracellular traps
ITM: intra-tumor microbiota
ICAM: intercellular adhesion molecule
GM: gut microbiota
IOM: immuno-onco-microbiome
OS: osteosarcoma
TP53: tumor protein 53
RB1: retinoblastoma protein
RECQL4: RecQ like helicase 4
BLM: bloom syndrome gene
WRN: Werner syndrome ATP-dependent helicase
EWS: Ewing sarcoma
CS: chondrosarcoma
EXT: exostosin glycosyltransferase
IDH: isocitrate dehydrogenase
CXCR4: C-X-C chemokine receptor 4
MMP: matrix metalloproteinase
CTGF: connective tissue growth factor
FGF5: fibroblast growth factor
IL: interleukin
OPN: osteopontin nuclear factor kappa-Β ligand (RANKL)
ET-1: endothelin-1
BMP: bone morphogenetic proteins
GDF15: growth differentiation factor 15,
MDSCs: myeloid-derived suppressor cells
ILCs: innate lymphoid cells
(TGF)-b: transforming growth factor
COX2: cyclooxygenase 2
IDO1: indoleamine 2,3-dioxygenase
PTHrP: parathyroid hormone-related protein
VEGF: vascular endothelial growth factor
MCSF: macrophage colony-stimulating factor
BMMs: bone-marrow-associated macrophages
CCL2: chemokine (C-C motif) ligand-2
DT: diphtheria toxin
GF: germ-free
ConvR: conventionally raised
IGF: insulin growth factor
TNF: tumor necrosis factor
SCFAs: short-chain fatty acids
HDACs: histone deacetylases
GPCR: G-protein-coupled receptors
NFaTc: nuclear factor of activated T cells
IBD: inflammatory bowel disease
CDT: cytolethal distending toxin
LCA: lithocholic acid
BV: bone volume
PMOB: post-menopausal-breast cancer

## CRediT authorship contribution statement

**Shreya Kapoor:** Writing – review & editing, Methodology, Investigation, Formal analysis, Data curation. **Muskan Gupta:** Writing – review & editing, Writing – original draft, Methodology, Investigation, Formal analysis, Data curation, Conceptualization. **Leena Sapra:** Writing – review & editing, Methodology, Investigation, Formal analysis, Conceptualization. **Taranjeet Kaur:** Writing – review & editing. **Rupesh K. Srivastava:** Writing – review & editing, Writing – original draft, Supervision, Project administration, Methodology, Investigation, Funding acquisition, Formal analysis, Conceptualization.

## Funding

This work was financially supported by projects: 10.13039/501100001407DBT (BT/PR41958/MED/97/524/2021), 10.13039/501100001411ICMR (61/05/2022-IMM/BMS) and an Intramural Grant (AC-73) sanctioned to RKS.

## Declaration of competing interest

The authors declare that they have no known competing financial interests or personal relationships that could have appeared to influence the work reported in this paper.

## Data Availability

No data was used for the research described in the article.
